# Nationalistic Media Obsession With Olympic Medal Counts: The Case of the 2020 Tokyo Olympic Games

**DOI:** 10.3389/fspor.2022.848071

**Published:** 2022-03-14

**Authors:** Steve Dittmore, Kibaek Kim

**Affiliations:** ^1^Department of Health, Human Performance and Recreation, University of Arkansas, Fayetteville, AR, United States; ^2^Department of Sports Industry, Korea Institute of Sport Science, Seoul, South Korea

**Keywords:** media, Olympic Games (OG), medal counts, framing, nationalism

## Abstract

Because Olympic medals are awarded to athletes representing an individual National Olympic Committee, it is natural for the media, and even the International Olympic Committee, to create a table indicating which nation has experienced the most athletic success. Problems, and even disagreements, arise when nations utilize different methods to count medals. The 2020 Tokyo Olympic Games, contested in 2021, provided a unique opportunity to observe how media organizations create a narrative around medal tables. American media outlets preferred to consistently show the United States at the top of the medal standings even though China had more gold medals for much of the Games' fortnight. Non-American media organizations took exception to that method of counting.

## Introduction

“Nationalism has been heralded as the nemesis that has plagued the modern Olympic Games since their inception in 1896,” (Toohey and Warning, 1981, p. 118).

When Avery Brundage became president of the International Olympic Committee, he inherited an increasingly nationalistic Olympic movement. The creation of new nation-states following World War II, many of them aligned with either capitalist or communist ideologies, presented a myriad of problems for Brundage as he sought to uphold the vision Pierre de Coubertin had for the Games. That vision centered on Coubertin's linguistic addition, “Olympism.”

Scholars regularly analyze and attempt to interpret what Olympism truly means. In their edited book on Olympism, (Seagrave and Chu, 1981) believed the concept “Aims at the harmonious development of the intellectual, moral and physical aspects of a human being through athletic competition.” Similarly, (Smirnov, 2000) concluded “Olympism had the idea to educate through the provision and encouragement of sport as one of the humanities. Its ideal was to transcend individual and national pride in competition.”

The growing ideological polarization and increased nationalism which greeted Brundage in 1952 contradicted the spirit of Olympism. Yet the structure and organization of the Olympic Games always competed with that spirit. Athletes compete for nations, not against one another. The parade of athletes is by nation, not by sport. Following the 1956 Melbourne Olympics, which featured a particularly violent water polo match between the Soviet Union and Hungary, Brundage would go so far as to write to IOC members encouraging the elimination of all team sports (Senn, 1999).

A prominent and overt manifestation of nationalism in the Olympics is the media-driven medal counts. Seemingly since American broadcast networks began purchasing Olympic media rights, the networks aggressively promoted medal counts for American athletes as a way to stimulate interest in its product, television broadcasts, by creating a rivalry between the United States and the rest of the world.

The rivalry angle worked well during the Cold War period, the 1960s−1980s, when the Olympics provided a literal playing field to argue which ideology was better, communism or capitalism. Eastern bloc countries regularly participated in state-sponsored doping as a means to grow medal counts and demonstrate a superior way of life. For the most part, it worked. Even in the era of tape-delayed broadcasts, Olympic programming garnered impressive audiences. The 1984 Olympic Games in Los Angeles garnered in excess of a 25 rating despite the absence of the Soviet Union and other Eastern bloc countries (Kaplan, 1984).

This “USA vs. them” approach was at the forefront of NBC's coverage during the opening ceremonies of the 2020 Olympic Games. After a lengthy patriotism-building introduction from Dwayne “The Rock” Johnson, NBC host Mike Tirico proclaimed definitively as Team USA entered the stadium, “613 athletes strong. Second largest Olympic team in U.S. History. No nation has won more medals at the Summer Olympics than the United States of America.” Given the changing geo-political landscape in the 1900s, that America has more medals than any other nation is not so surprising. The People's Republic of China did not enter the Olympic arena until 1952 and Russia has competed under the Russian flag, the Soviet Union, the Unified Team, and the ROC.

But, like most statistics, Olympic medal counts can be viewed from multiple angles. Which is better: most gold medals, or most overall medals? NBC had certainly emphasized the overall angle during the Opening Ceremony broadcast. Team USA had comfortably garnered both titles during the 2016 Rio de Janeiro Games, with 121 total medals, 46 of which were gold. Great Britain finished second with 27 gold medals while China was second with 71 overall medals.

The U.S. media's attempt to play up the most patriotic narrative began to unfold early in Tokyo as Team USA failed to earn a medal in any sport on Day One of the Olympics for the first time since the 1972 Munich Games, nearly a half-century ago. As Yahoo's Jack Baer qualified, the Day One sports which included archery, fencing, judo, shooting, and taekwondo, were not “ones that typically power the U.S. medal count, like swimming, track & field, gymnastics and basketball. There wasn't even an American entered in the judo and taekwondo events” (Baer, 2021).

NBC used political analyst Steve Kornacki, famous for crunching election night numbers on his big board, to help break down the medal tally. During a morning interview on MSNBC on July 29, 2021, nearly a week into the Games, Kornacki noted, the United States was second behind China with 14 gold medals, but a graphic showed Team USA at the top with 38 total medals, seven in front of China.

As if to build early momentum for the narrative, Kornacki proclaimed, “There is a bit of a streak on the line here for the United States. The last two Summer Games before this they ended up finishing with the most gold medals. It was a runaway back in 2016. We still have plenty of time left in these games, so we'll see if the U.S. can keep that streak going.

The other streak that is still alive for the U.S. is that they have had the most medals of any country at the Summer Games for six straight Olympic games now,” (Steve Kornacki's Olympic Medal Update, 2021).

During NBC's primetime coverage of the Games on August 1, Kornacki appeared at his big board, breaking down the U.S. medal situation. “How about the U.S. vs. the rest of the world? What does the medal race look like? With that 60th medal the U.S. just won, they extend the lead over China. Two streaks on the line here in Tokyo for the U.S. For six straight Olympic games, dating back to the 1990s, the U.S. Has finished with the most overall medals and they seem to be on course for that right now. Two straight Olympics, the U.S. Has finished with the most gold medals. That one they have a little work to do compared to China. 24-20 China,” Kornacki said.

The graphic on his board and NBC's airwaves, however, positioned the United States on top, despite trailing China in the gold medal count. It was this narrative, the U.S. on top of medal standings despite having fewer gold medals than China, that created controversy but also served as a benefit for both countries' media outlets.

NBC chairman Mark Lazarus had said in 2017, “I actually believe it should be based on total medals as opposed to gold only because I don't think you should be diminishing the accomplishments of the silver and bronze medalists who have achieved something extraordinary, either personally or as a team. So that would be my take on it. But we've never formally discussed how we present it,” (Billings et al., 2017, p. 119).

## American Media Criticized

At 9:15 a.m. EDT on August 2, 2021, the day after Kornacki's graphic in primetime, the *New York Times* tweeted a Medal Count graphic showing the United States ahead with 64 total medals. The problem was in the ranking by gold medal where the U.S. trailed China, 29 to 22. Social media backlash was swift with many individuals pointing out the IOC structures its medals table by gold medals won, though the organization allows users to sort its online medal table by a number of criteria. As observers noted, this contradicted the *Times'* own reporting at the conclusion of the 2016 Olympic Games in Rio de Janeiro in which Great Britain is listed second with 27 gold medals and China, with the second-most overall medals, is listed third (Rio Olympics Medals, 2016). The U.S. finished first in both gold and overall medals in 2016.

NBC also doubled-down on the narrative during its primetime coverage on August 2, 2021, showing a graphic similar to the Times with the United States on top with 64 overall medals, two ahead of China which held the lead in gold medals, 29–22. Tirico remarked, as the 10:00 p.m. EDT hour began, “It all adds into the medal count, which is what we're looking at as we started this Tuesday in Tokyo. The United States still in the overall lead. China won five gold medals on Monday, so they've extended the advantage there.”

The next day, August 3, 2021 at 6:15 a.m. EDT, the *Times* tweeted a different graphic showing countries ranked by gold medals won to that point. China was first with the United States second. The damage was done. To the critics, this was blatant nationalistic homerism by the “Gray Lady.” As Graeme Massie reported in *The Independent* newspaper in the United Kingdom, under the headline, “American media outlets criticized over Team USA bias in Olympic medal table,” the U.S. outlets were “misrepresenting” the results (Massie, 2021).

Some, not all, American media picked up on the controversy, led by Yahoo.com's Dan Wetzel who opined on August 4, “China is kicking the United States' tail (at least for now) in these Olympics, although you wouldn't know it if you just scanned the medal tables in the American media. In an unexplained yet (apparently) nationally accepted counting method, Americans tally the standings not by what country wins the most golds, but what country wins the most total medals,” (Wetzel, 2021).

Wetzel continued, “The rest of the world favors gold over everything. That's how the International Olympic Committee tallies it. Same with the medal standing on the Toyko 2020 website. It's good enough for media companies all over the world, just not in the U.S. apparently.”

That night, August 4, 2021, NBC seemingly began making excuses for the U.S. team's performance. Following the broadcast of an American gold medal in the shot put, NBC showed the medal standings with the U.S. still on top in overall medals, 83–71, but trailing in golds, 32–27. Said Tirico as the graphic was being aired, “gold and silver for the United States in the shot put adds to the medal count where the U.S. continues to maintain the overall edge by a dozen up to the moment over China, five behind China in terms of the conversation for gold medals at these games. The medal count, of course, becomes one of the official records when we look back at Olympic Games, but with these Olympics, which of course coming in were destined to be remembered a little bit differently, one thing that's become apparent is that with the best athletes, who they are and how they got here is more important than what they do here.”

Tirico continued talking about Simone Biles, Michael Phelps, and Noah Lyles and their public discussion of struggles with mental health and depression. Tirico commented, “A lot more story to write for (Lyles) and for all the athletes here, far beyond what that medal count that we share might tell you years from now.”

Was NBC attempting to shift the narrative away from medal tables and which country had the most gold medals and toward an athlete's mental health? And, if so, why? Did the network fear Team USA would not live up to expectations?

In the end, the United States won both medal counts at the Tokyo Olympics, most overall medals (113 to China's 88) and most gold medals (39 to China's 38), thanks to the U.S. women's volleyball team gold medal on the final day of competition (Shivaram and del Barco, 2021).

That night, during NBC's broadcast of the Closing Ceremony, commentator Johnny Weir remarked, “Not only did (the United States) top the overall medal count but also the gold medal count at this games, so truly a spectacular performance.”

## Chinese Media Pads Medal Count

On the eve of the final day of competition, China's state-run CCTV apparently took exception with the United States method of determining which country won the most medals, decrying the U.S. approach as “ranking the U.S. first based on ‘American standards,”' (Everington, 2021). At the time, China had more gold medals than the United States. In the days which followed, reports indicated Chinese state media had manipulated medal counts by including 12 medals won by athletes representing Taiwan, which competes as Chinese Taipei, and six medals won by athletes representing Hong Kong, both of which have independent National Olympic Committees sanctioned by the IOC. The new total, circulated on the Chinese social media platform Weibo, showed China with 42 gold medals and 106 total medals. It was accompanied with the caption, “Congratulations to the Chinese delegation for ranking first in gold medals and the total number of points” (Steinbuch, 2021). Using a point system of 3 for a gold, 2 for a silver, and 1 for a bronze, the new total would have China with 227 points and the United States with 225 points.

## British Media Suggest Alternative Medal Table

While the United States extended its streak to a seventh straight Summer Olympics in which it dominated the medal count, the BBC in the United Kingdom sought to frame the narrative differently. Consulting “economists and data nerds,” writer Robin Levinson-King envisioned a table in which medals were ranked by population and wealth. “Some countries over-perform, given their population size. The BBC came up with an alternative ranking, which looked at the number of medals won per million people,” (Levinson-King, 2021).

By population, the tiny European nation of San Marino, with its overall population of 33,000 and three total medals in Tokyo, came out on top. The United States ranked 60th by population. In considering wealth, as measured by GDP per capita, China emerged on top followed by the ROC and Kenya. The United States would have finished 15th.

“If a country is very poor, it won't have the resources to convert that potential into actual ability to compete on a world stage,” David Forrest, an economist at the University of Liverpool told the BBC. “They've got to have the ability to participate in sport in the first place. For example, they might have a great natural ability in swimming that is waiting to be developed—but actually there won't be any swimming pools,” (Levinson-King, 2021).

Levinson-King also noted the relationship between countries with lower GDPs, called “poorer countries,” and lower-cost sports such as wrestling and track. The inverse was also discussed in which higher GDP countries tend to out-perform in higher-cost sports such as equestrian and sailing (Levinson-King, 2021).

## Conclusion

Even if the International Olympic Committee adjusts its program to eliminate, or at least reduce, the influence of nationalistic rituals such as the parade of nations and playing of national anthems, the Games will continue to be subject to nationalism and the challenges which accompany it. Media organizations emphasize broadcasting events featuring athletes from their countries because audiences appreciate it. This is particularly true of American television.

In their 2017 book on Olympic broadcasting, Billings, Angelini, and MacArthur interviewed NBC chairman Mark Lazarus who stated emphatically, “We don't say ‘we' and we don't say ‘us.' We say the Americans or Team USA. We also have a country that's a melting pot and there's a lot of people here cheering for other folks,” (Billings et al., 2017, p. 117).

However, as Billings et al. (2017) concluded in their study of 20 years' worth of Olympic telecasts, that philosophy does not match practice. “While Team USA has enjoyed considerable success, the amount of network emphasis on American athletes is disproportionate to their success,” (p. 98-99). The authors observed that American athletes received 52.3 percent of mentions in NBC's 2016 broadcast, roughly 3.5–4.2 times their medal count (Billings et al., 2017).

Billings (2008) was one of the early scholars to suggest a medals table, as a measure of overall achievement, could lead to a perception that a single nation “won” the Olympics. However, as the British economists noted in 2021, overall medal count is just one metric to evaluate a nation's performance at the Olympic Games.

NBC's Mike Tirico was correct. The medal count does become part of the permanent record of an Olympic Games. It is a documentation of the athletes and their performance. It does not need to be, as was observed in 2021, a proxy for a nation's perceived superiority.

## Data Availability Statement

The raw data supporting the conclusions of this article will be made available by the authors, without undue reservation.

## Author Contributions

SD writing and researching manuscript. KK content analysis and coding of broadcasts. Both authors contributed to the article and approved the submitted version.

## Funding

Research support for this article provided by the University of Arkansas Center for Communication Research. Research support for this article provided by the Open Access Publishing Fund administered through the University of Arkansas Libraries.

## Conflict of Interest

The authors declare that the research was conducted in the absence of any commercial or financial relationships that could be construed as a potential conflict of interest.

## Publisher's Note

All claims expressed in this article are solely those of the authors and do not necessarily represent those of their affiliated organizations, or those of the publisher, the editors and the reviewers. Any product that may be evaluated in this article, or claim that may be made by its manufacturer, is not guaranteed or endorsed by the publisher.
